# A Broad Dual-Band Implantable Antenna for RF Energy Harvesting and Data Transmitting

**DOI:** 10.3390/mi13040563

**Published:** 2022-03-31

**Authors:** Yi Fan, Xiongying Liu, Chao Xu

**Affiliations:** 1 School of Electronics and Information, Guangdong Polytechnic Normal University, Guangzhou 510641, China; hnanfy@163.com; 2 School of Electronic and Information Engineering, South China University of Technology, Guangzhou 510641, China; students_china@163.com

**Keywords:** dual bands, implantable antenna, rectenna, rectifier, RF energy harvesting, wide band, wireless power transfer

## Abstract

An implantable antenna, operating at the dual Industrial, Scientific, and Medical (ISM) bands of 902–928 MHz and 2.4–2.48 GHz, is presented for RF energy harvesting and data transmission. By introducing multiple radiating branches and etching a C-shaped slot, multiple resonant frequencies were generated to produce the wide dual bands. The proposed antenna has compact dimensions of 7.9 × 7.7 × 1.27 mm^3^. The simulated impedance bandwidths of the antenna are 0.67–1.05 GHz (44.2%) and 2.11–2.96 GHz (33.5%), and the peak gains are −28.9 dBi and −29.5 dBi, respectively. The lower band can be applied in RF energy harvesting, while the upper band is designed for data transmission with external medical equipment. Furthermore, to convert RF power into DC power, in RF energy harvesting, a voltage-doubled rectifier was positioned under the ground plane of the proposed antenna. The rectifier circuit can achieve a maximum conversion efficiency of 52% at an input power of 5 dBm. Furthermore, the integrated scheme of the implantable rectenna was fabricated and the numerical performance of the wireless power transfer was verified with the measurement results.

## 1. Introduction

Implantable medical devices (IMDs), which can monitor physiological data inside the human body in real time and treat diseases remotely, are playing an increasingly significant role in telemedicine [1]. These IMDs include blood pressure monitors [2], cochlear implants [3], and implantable pacemakers [4], and others. However, the working lives of IMDs are limited by their batteries [5,6]. Due to the limited space of IMDs, the capacity of their batteries is not large enough. Therefore, if the batteries of IMDs run out and need to be replaced, patients suffer the pain associated with multiple surgeries. To address the above issues, wireless power transfer (WPT) technology in medical systems can be adopted [7]. 

In the WPT of medical systems, the power of the RF energy source is usually transferred to the IMDs by means of inductive coupling [8,9,10] or microwave radiation [11,12]. The application of inductive coupling to a WPT system is easily affected by the angular misalignment between the transceiver coils and suitable for short-distance energy transmission. However, the application of microwave radiation mode has higher robustness and less sensitivity to changes in antenna position and direction. In addition, the implantable antenna used in microwave radiation has a smaller size and longer transmission distance. Comparing these two methods, WPT systems that adopt microwave radiation are more suited to IMDs.

Whether they are used for RF energy harvesting or data transmission in IMDs, the implantable antenna, as an important component in electromagnetic energy conversion, is indispensable. According to various scenarios, researchers have designed several types of implantable antenna, including circularly polarized (CP) antennas [13,14,15], multi-input and multi-output (MIMO) antennas [16,17], conformal antennas [18,19], and differentially fed antennas [20,21]. However, these antennas are only investigated for their performance of the data transmission function; RF energy harvesting is not taken into account in these schemes. Therefore, some implantable antennas with functions of wireless energy harvesting and data transmission were proposed in [22,23,24]. In [22], a dual-band implantable rectenna, which used a matching layer on the arm to enhance the wireless power link, was introduced for data transmission and RF energy harvesting. In [23], a circular dual-band implantable antenna with a radiating slot patch was proposed. In order to enhance the power transmission link, an additional external metallic reflector was placed behind the human arm. A self-diplexing implantable antenna with two ports with a minimized size was reported in [24] for RF energy harvesting and data transmission. However, the impedance bandwidths of the above antennas are relatively narrow, due to the complexity of human tissues; the antennas applied in IMDs should have a broad bandwidth to avoid deviation from the desired band.

In this work, a broad dual-band implantable antenna for RF energy harvesting and data transmission is investigated. By introducing multiple radiating branches and etching a C-shaped slot, multiple resonant frequencies were generated to achieve dual and broad bands. Furthermore, the dimensions of the proposed antenna were minimized to 7.9 × 7.7 × 1.27 mm^3^. The impedance bandwidths of the proposed antenna can cover the 915-MHz and 2.45-GHz ISM bands. The 915-MHz ISM band can be applied in RF energy harvesting, and the 2.45-GHz ISM band can be used for data transmission. Furthermore, to be applied in WPT, the rectifier circuit that converts RF power into DC power is designed under the ground plane. Furthermore, the integrated structure of the implantable rectenna for RF energy harvesting was measured and is presented here.

## 2. Antenna Design and Discussion

### 2.1. Antenna Design

**Figure 1:**
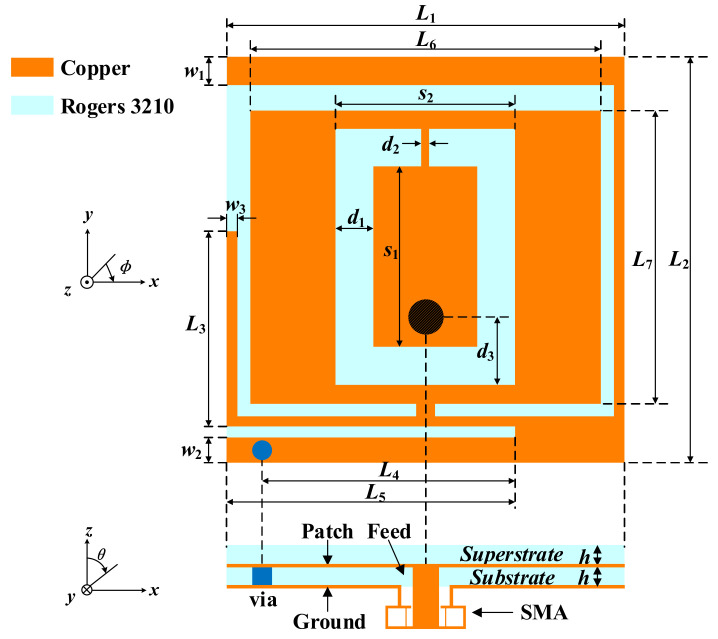
Geometry of the proposed implantable antenna.

The configuration of the proposed implantable antenna is displayed in Figure 1. The optimized dimensions with the electromagnetic numerical simulation software ANSYS HFSS v.18 are listed in Table 1. The radiation patch is printed on the substrate of Rogers 3210 with a relative permittivity of *ε*_r_ = 10.2 and a loss tangent of tan *δ* = 0.003; it is covered with a superstrate of the same material, which can isolate the radiation patch from the surrounding human tissues and plays a buffer role between the conducting radiation patch and lossy human tissues.

**Figure 2:**
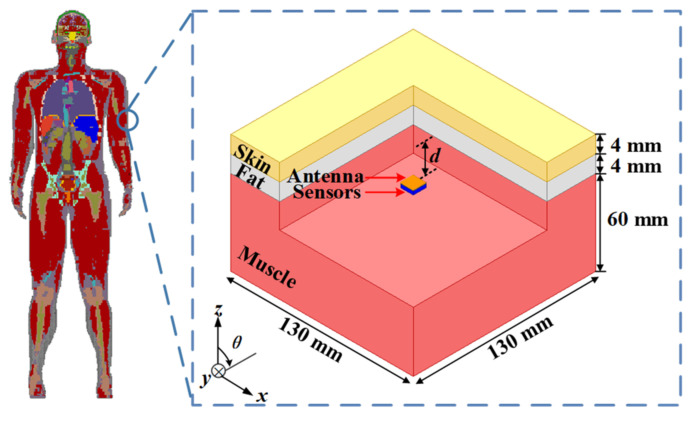
Simulation environment of the three-layer phantom model.

In the simulation analysis, the three-layer phantom in Figure 2 was employed to evaluate the performance of the proposed antenna. The thicknesses of the skin, fat, and muscle layers were 4 mm, 4 mm, and 60 mm, respectively. The proposed antenna was implanted in the muscle layer. The distance from the antenna to the bottom of the fat was 4 mm, and the total depth of the implantation was 12 mm. In the simulation of the dual-band antenna, the dielectric properties of human tissues, such as relative permittivity and loss tangent, should be set as the values at the center frequency in the two target bands [25]. The detailed dielectric properties are listed in Table 2.

**Figure 3:**
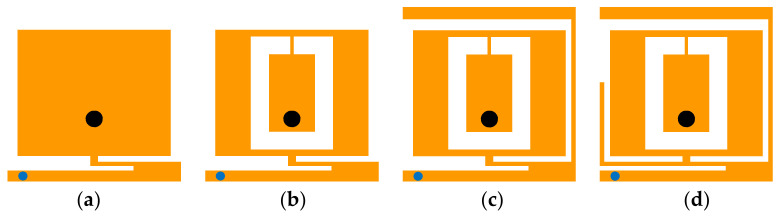
Evolution of the proposed antenna topology in (**a**) Case 1, (**b**) Case 2, (**c**) Case 3, and (**d**) Case 4.

**Figure 4:**
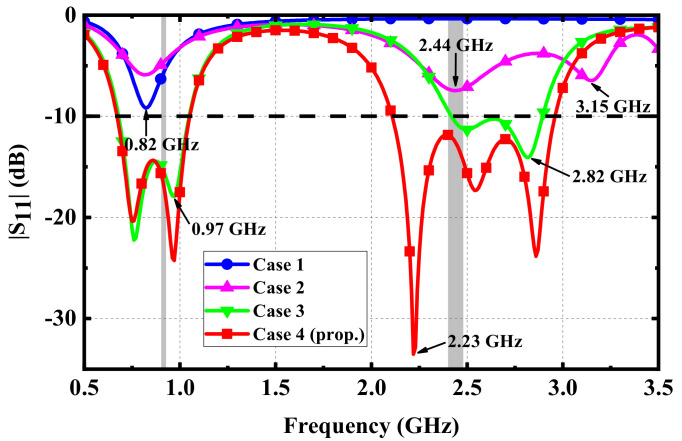
Simulated results of different cases.

The detailed steps of the simulation are shown in Figure 3, and the simulated results for different cases are analyzed in Figure 4. In Case 1, to achieve miniaturization, a rectangular patch with a metal branch and via was introduced to form a PIFA, resulting in a resonant frequency at 0.82 GHz, near the 915-MHz ISM band. To achieve the performance of the dual band, a C-shaped slot was etched in the middle of the rectangular patch to form multiple independent current paths in Case 2, which added two resonant frequencies at 2.44 GHz and 3.15 GHz, near the 2.45-GHz ISM band. In Case 3, a long L-shaped metal branch on the right side was loaded to extend the bandwidth of the antenna at the 915-MHz ISM band, which excited the 0.25*λ* monopole mode at 0.97 GHz. The resonant frequency at 0.97 GHz was close to the original resonant frequency in Case 1, which expanded the bandwidth at the low-frequency band. Meanwhile, the long L-shaped metal branch extended the current path at 3.15 GHz in Case 2, which reduced the resonant frequency to 2.82 GHz and improved the impedance matching. To further expand the bandwidth at the 2.45-GHz ISM band, a L-shaped metal branch on the left side was introduced in Case 4, which excited the 0.25 λ monopole mode at 2.23 GHz. Therefore, the three resonant frequencies excited at the upper frequency band were close to each other, which expanded the bandwidth of the proposed antenna at the 2.45-GHz ISM band. As a result, wide bandwidths in 915-MHz ISM and 2.45-GHz ISM were produced.

**Figure 5:**
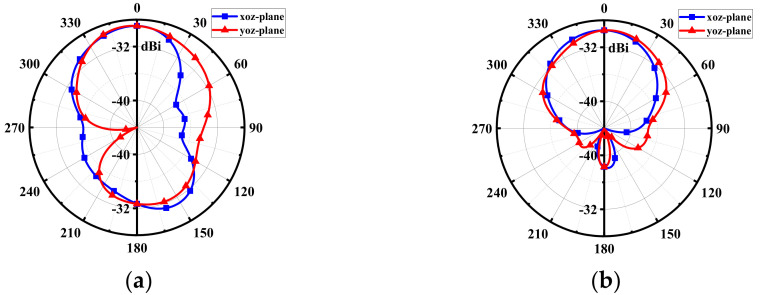
Simulated radiation patterns at (**a**) 915 MHz and (**b**) 2.45 GHz.

Figure 5 shows the radiation patterns of the proposed antenna at 915 MHz and 2.45 GHz, with peak gains of −28.9 dBi and −29.5 dBi, respectively. Due to the effect of the human tissues, the back lobe of the radiation pattern at 915 MHz is a little large. Nevertheless, since the 915-MHz ISM band is used for receiving RF energy in WPT, there can be no backward radiation to the human body. The radiation pattern at 2.45 GHz has good orientation, making it very suitable for data transmission with external medical devices.

### 2.2. Sensitivity Analysis

**Figure 6:**
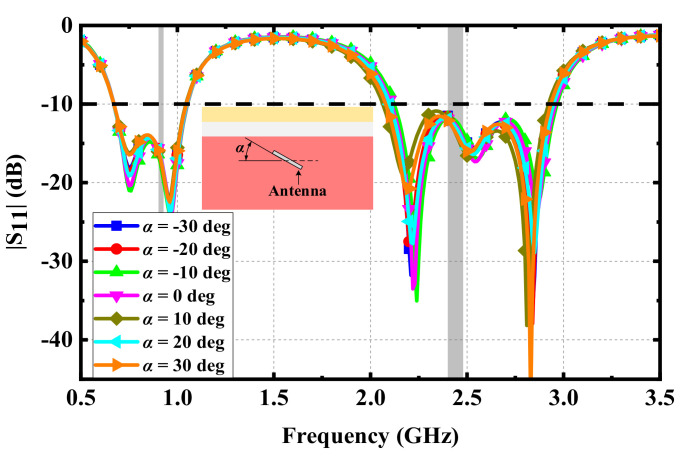
Simulated |S_11_| varying with the incline angles of implantation.

**Figure 7:**
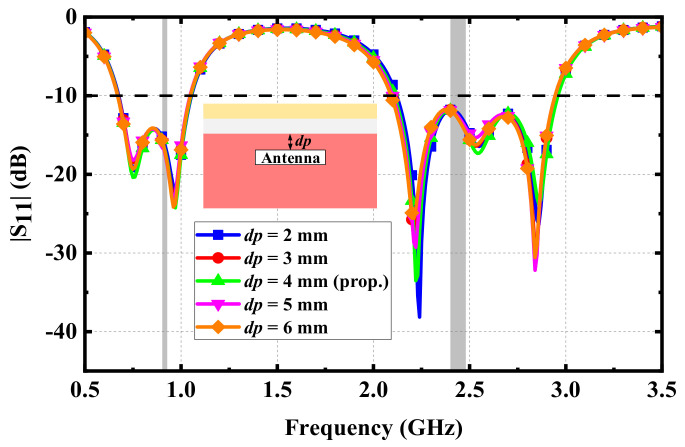
Simulated |S_11_| varying with the implanted depths.

**Figure 8:**
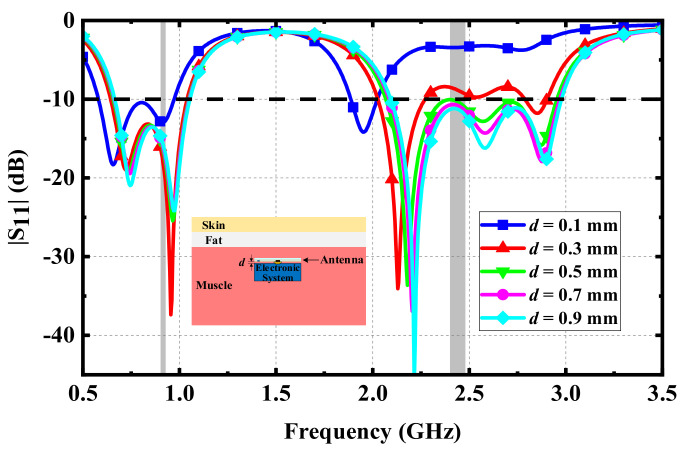
Simulated |S_11_| varying with the distance between the proposed antenna and the electrical system.

**Figure 9:**
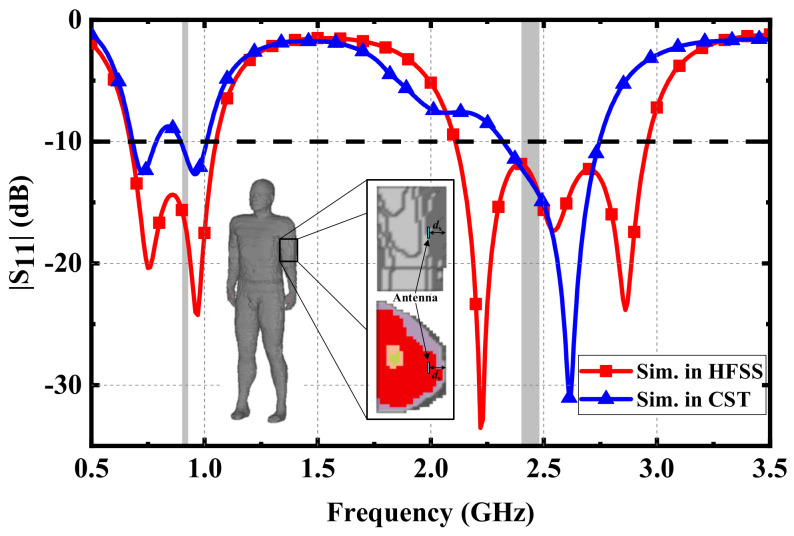
Comparison of the simulated |S_11_| in the three-layer phantom of HFSS and the Hugo phantom of CST.

- Effects of variations in implantation: During the surgical implantation of IMDs into human tissues, the position of the proposed antenna may be inclined. Moreover, human tissues are not high-hardness mediums; they are easily affected by extrusion and other unexpected factors in daily life, resulting in changes in the incline angles of the proposed antenna. Therefore, the possible impact on the antenna should be analyzed. Figure 6 shows the effect of different incline angles on the |S_11_| of the proposed antenna. Since the change in the incline angles did not change the dielectric properties of the human tissues around the antenna, the |S_11_| of the proposed antenna was basically unaffected. Hence, the proposed antenna can maintain good performance. Following the same considerations, the performance of the proposed antenna at different implanted depths was evaluated, as shown in Figure 7. The resonant frequencies of the proposed antenna at different implanted depths also remained unchanged, and the effects of the impedance matching at each resonant frequency were very small, indicating that the proposed antenna has good robustness.

- Effects of electrical components in the IMD: Implantable antennas do not work as single devices implanted in human tissues. That is, there are many other electronic components in IMDs. For example, in the RF energy harvesting system, rectifiers are usually connected with the proposed antenna. Thus, it is necessary to analyze the influence of other electronic components on the proposed antenna. Since these electronic components are usually made of metal materials, they can be analyzed by an equivalent analysis of metal blocks during the simulation. Therefore, a metal block with the same length and width as the proposed antenna was placed under the antenna to analyze the influence of the metal block on the antenna with a distance *d*. It can be seen from Figure 8 that with the gradual decrease in distance *d*, the impedance matching of three resonant frequencies in the upper frequency band became worse, while it was not affected in the lower frequency band. Furthermore, the simulated results show that the proposed antenna had better impedance matching and wider impedance bandwidth when the distance between the electronic system and the proposed antenna was more than 0.5 mm.

- Effects of different simulation phantoms: When the antenna was originally designed, a simple three-layer human phantom was employed to analyze and optimize the dimensions of the antenna, which can reduce the time of simulation and simplify the design process. However, in a real human environment, different types of tissues and organs are complex and unevenly distributed. Hence, it is necessary to further evaluate the performance of the proposed antenna in a high-precision human phantom. The Hugo human phantom shown in the inset of Figure 9 was employed, and the proposed antenna was implanted in the muscle tissue of the arm. Figure 9 shows the comparison between the optimized results of the Hugo human phantom in CST and those of the three-layer human phantom in HFSS. Due to the inhomogeneity and complexity of human tissues, the impedance matching of the two resonant frequencies in the lower frequency band became worse, resulting in the |S11| in the middle part of the lower frequency band being higher than −10 dB. Furthermore, the impedance bandwidths with |S11| less than −10 dB in the lower frequency band were 0.68–0.79 GHz and 0.89–1.01 GHz. Among the three resonant frequencies in the upper frequency band, the impedance matching of one resonant frequency was worse, resulting in the impedance bandwidth narrowing to 2.32–2.74 GHz in the upper frequency band. Although the impedance bandwidths of the proposed antenna at two frequency bands were narrower than those simulated in HFSS, the antenna could still cover the dual ISM bands of 902–928 MHz and 2.4–2.48 GHz, which verified the performance of the proposed antenna. Furthermore, to achieve the best performance in complex human environments, the impedance matching of the proposed antenna can be further optimized through the parameter analysis described above.

**Figure 10:**
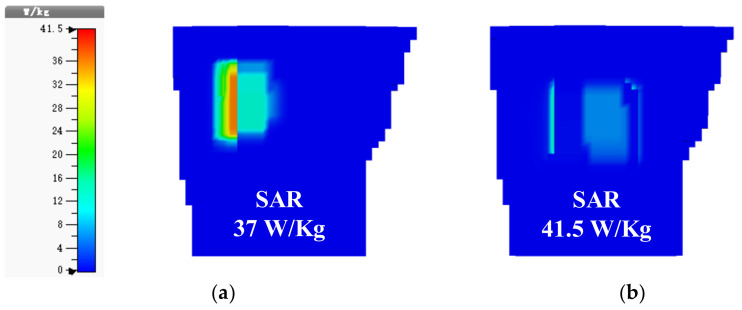
Simulated SAR distributions for 10-gram tissues at (**a**) 915 MHz and (**b**) 2.45 GHz.

In addition, because the implantable antenna is placed in the human body, the electromagnetic field generated by the antenna interacts with that of the human body to produce biological effects that interfere with the electromagnetic environment in the organism. Thus, the human body manifests symptoms such as headache, memory loss, and heart failure, and the skin may also burn. These damages to the human body are long-term and chronic. The specific absorption rate (SAR), defined as the RF power absorbed per unit of mass of an object (in W/kg), is one important index, reflecting whether an implantable antenna radiates safely. Therefore, it is necessary to evaluate the SAR of the proposed antenna in the Hugo human phantom. Figure 10 shows the simulated SAR distributions for 10-gram tissues at 915 MHz and 2.45 GHz with an input power of 1 W. The maximum SAR values at two frequencies were 37 W/kg and 41.5 W/kg, respectively; according to IEEE C95.1-2005 [26], the maximum value of the average SAR of any 10-gram tissue should be limited to 2 W/kg. As a result, the simulated results show that the maximum input power of the proposed antenna at 915 MHz and 2.45 GHz should not exceed 54.1 mW and 48.2 mW in order to ensure radiation safety.

### 2.3. Measurement

**Figure 11:**
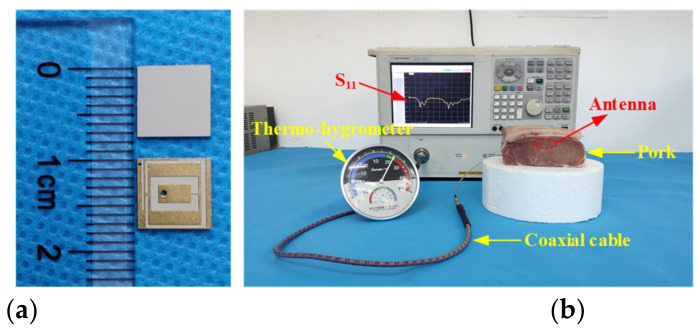
Measurement of the proposed antenna: (**a**) Fabricated antenna and (**b**) measurement setup.

To test the performance of implantable antennas conveniently, many scholars simply mimic skin or muscle via tissue-equivalent fluid. Although this method can easily achieve the permittivity and conductivity of skin or muscle at a single frequency, it is not suitable for the measurement of multiband implantable antennas. For multiband implantable antennas, animal tissues are the preferred measurement environment due to the fact that the electrical characteristics of animal tissues are frequency-dependent and similar to human tissues. Figure 11 shows the schematic diagram of a measurement employing the animal tissue of fresh pork. The proposed antenna implanted in the pork was connected to the vector network analyzer through the coaxial cable.

**Figure 12:**
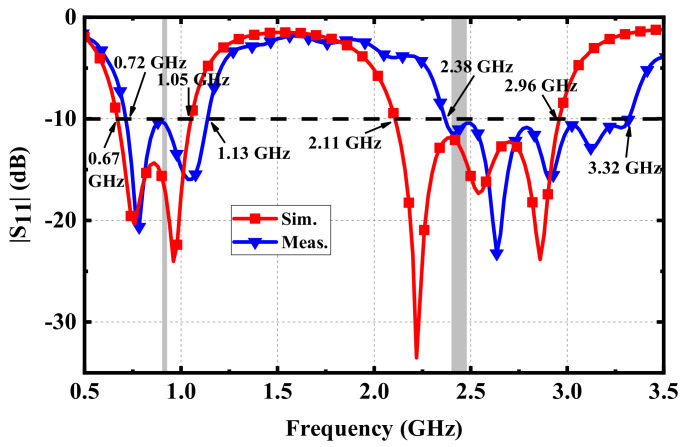
Comparison between simulation and measurement.

It can be seen from the measurement result in Figure 12 that the measured |S_11_| in the lower-frequency band was consistent with the simulated result, while the frequency offset in the upper frequency band was obvious. The reason for this may be that the gap between the radiation patch and the superstrate was generated unintentionally during the manual welding of the feed. This can be avoided by using precision processing equipment. Nevertheless, the measured |S_11_|of the proposed antenna can still cover the ISM bands of 902–928 MHz and 2.4–2.48 GHz. The measured relative bandwidths were 44.3% (0.72–1.13 GHz) and 33.0% (2.38–3.32 GHz) in the two frequency bands, respectively, both of which were close to the relative bandwidths in the simulation.

## 3. Rectenna Design

### 3.1. Rectifier Design

In RF energy-harvesting systems using microwave radiation, the function of the implantable antenna is to receive the RF power from the energy source. Furthermore, the high-frequency rectifier circuit, which also plays a critical role in RF energy-harvesting systems, can convert the RF energy into a DC signal, powering or charging the IMDs. Therefore, it is necessary to design a rectifier circuit in the working frequency band of the proposed antenna to achieve the conversion and utilization of RF power. 

**Figure 13:**
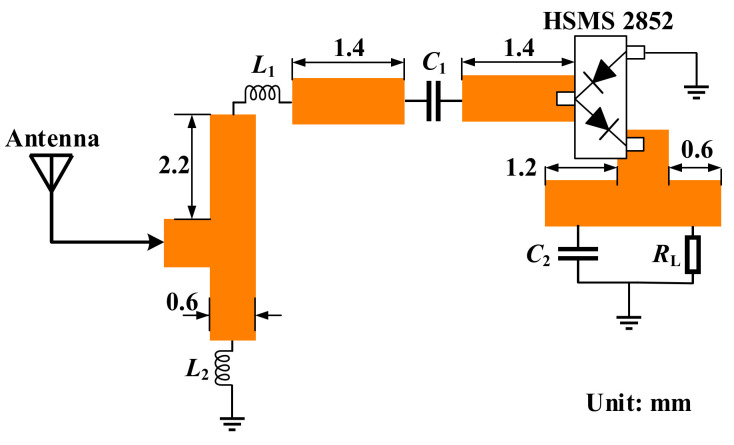
Schematic of the rectifier circuit. The parameters are: *L*_1_ = 18 nH, *L*_2_ = 5.1 nH, *C*_1_ = 100 pF, *C*_2_ = 330 pF, and *R*_L_ = 4.3 kΩ.

**Figure 14:**
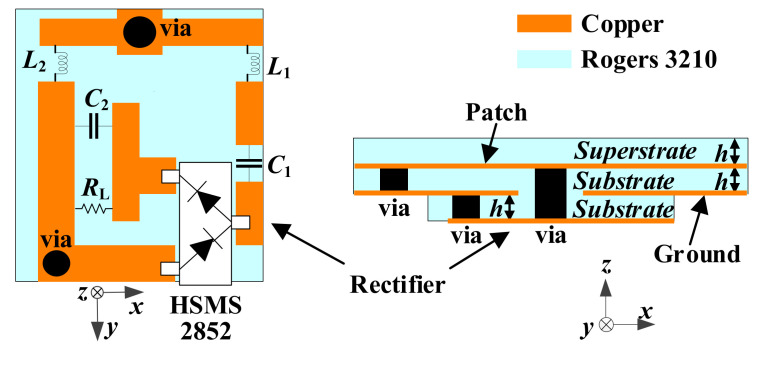
Configuration for integrating the rectifier circuit as a rectenna.

**Figure 15:**
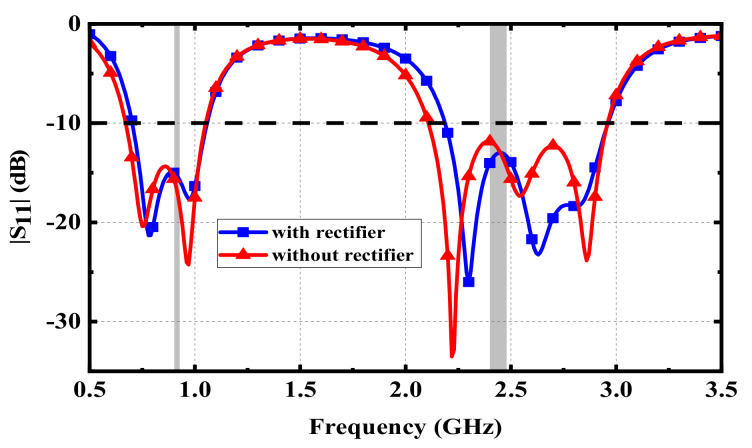
Effect of adding the rectifier layer on the proposed antenna.

In the proposed dual-band implantable antenna, the 915-MHz ISM band with lower path loss was applied to implement RF energy harvesting. In this study, the schematic diagram of the rectifier circuit was designed in the Keysight ADS, as shown in Figure 13. The output DC voltage could be increased by employing the voltage-doubled rectifier circuit, which was mainly composed of a Schottky diode, HSMS 2852, impedance-matching elements of *L*_1_ and *L*_2_, a DC isolation capacitor of *C*_1_, a DC filter capacitor of *C*_2_, and a load of *R*_L_. The HSMS 2852 Schottky diode was selected because of its low-forward-bias voltage and high cut-off frequency. Furthermore, the lower forward voltage drop made it possible to achieve rectification at low input voltage bias, which is conducive to low power consumption. The performance of the rectifier can be evaluated by the RF-to-DC conversion efficiency as

(1) $$\eta (\%)=\frac{P_{\mathrm{DC}}}{P_{R}}\times 100\%=\frac{V_{\mathrm{DC}}^{2}}{R_{L}\times P_{R}}\times 100\%$$

where *P*_R_ represents the RF power received by the antenna, and *P*_DC_ denotes the DC power output by the rectifier circuit. In addition, to be integrated with the implantable antenna as a whole, a substrate of Rogers 3210 was added under the ground of the proposed antenna. The rectifier circuit was placed on the back of the substrate, forming a rectenna. Figure 14 illustrates the optimized structure of the proposed rectenna. The rectifier circuit can be connected with the radiation patch and the ground of the proposed antenna through two vias. Figure 15 shows the effect on the proposed antenna after adding the rectifier circuit. The effects of inductors and capacitors are replaced by ideal conductors in HFSS. Since the substrate layer of the rectifier circuit is composed of Rogers 3210, its relative permittivity is lower than that of human muscle. Therefore, after adding the substrate layer of rectifier circuit, the equivalent permittivity around the proposed antenna becomes lower, which makes the resonant frequencies shift to higher frequencies. A slight offset has little effect on the antenna due to the wide bandwidth and the full ground, guaranteeing that the proposed antenna has good robustness.

**Figure 16:**
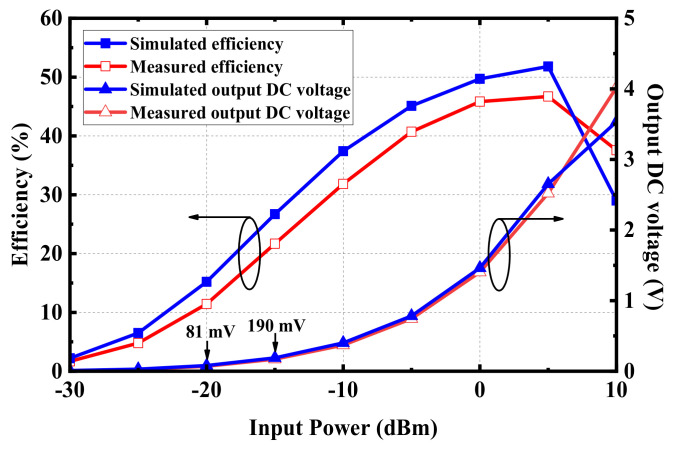
RF-to-DC conversion efficiency and output DC voltage of the rectifier.

To evaluate the performance of the rectifier circuit, the rectifier circuit in Figure 14 was fabricated and tested. In the process of measurement, the power source was transmitted into the rectifier circuit with different RF power. The DC voltage of *R*_L_ can be measured by a voltmeter, and the RF-to-DC conversion efficiency can be calculated by Formula (1). Figure 16 shows the simulated and measured conversion efficiency and the DC voltage of *R*_L_. When the input power is 5 dBm, the rectifier circuit can achieve a maximum simulated conversion efficiency of 52% and a measured conversion efficiency of 45%. Furthermore, the rectifier circuit shows good performance at low input power, which is suitable for IMDs with low received power.

### 3.2. Evaluation of Received Power

Before measuring the performance of the implantable rectenna, the RF power that the implantable antenna may theoretically receive can be evaluated as

(2) $$P_{r}(dB)=P_{t}+G_{t}-L_{tf}-L_{f}+G_{r}-L_{rf}$$

(3) $$L_{f}(dB)=10\mathrm{lg}{\left(4\pi d/\lambda \right)}^{2}$$

where *P*_r_ and *P*_t_ are the received power and the transmitting power, respectively; *G*_r_ and *G*_t_ are the maximum gains of the implantable antenna and the external transmitting antenna, respectively; *L*_f_ is the path loss in free space; and *L*_tf_ and *L*_rf_ are the mismatch loss of transmission and reception, respectively.

**Figure 17:**
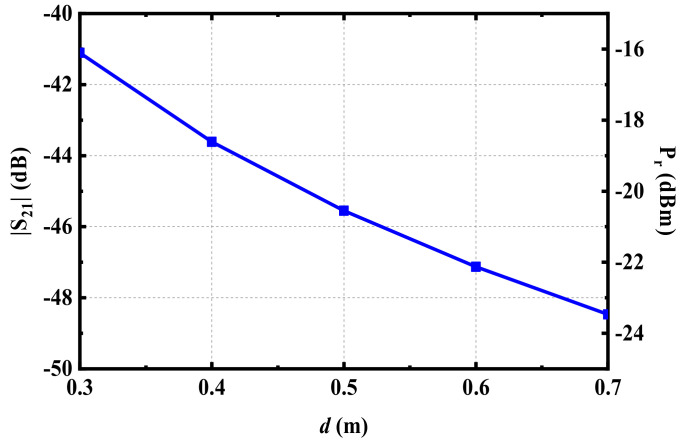
Evaluation of transmission efficiency and received power.

The maximum gain (*G*_r_) of the proposed implantable antenna at 915 MHz is −28.9 dBi. If a panel antenna with a gain (*G*_t_) of 9 dBi is employed to transmit RF power of 25 dBm, assuming that the transceiver mismatch loss is ignored under ideal conditions, that is, that *L*_tf_ and *L*_rf_ are zero, the RF power of the proposed antenna and the S_21_ can be calculated, as given in Figure 17 by Formula (2). The calculated results in Figure 17 are the maximum RF power that the proposed antenna can receive in theory, but in actual situations, the received RF power would be affected by many other factors. It can be seen from Figure 17 that the closer the distance between the transmitting and receiving antennas, or the greater the transmitting power, the higher the power received by the proposed implantable antenna. However, too close a distance or a transmitting power that is too high may affect the radiation safety of the human body. Therefore, before employing the rectenna to collect RF energy, it is necessary to evaluate its radiation safety.

### 3.3. Radiation Safety

When IMDs receive RF power, it is essential to determine the power that the RF energy source can radiate to the surface of human tissues within radiation safety limits. According to the limit on the maximum allowable exposure (MPE), the power flux density generated by the radiating antenna at 915 MHz should be less than 6 w/m^2^ [27]. The power flux density *S*(*x*) from the radiating antenna at a distance of *x* can be calculated as


(4) $$S(x)=\frac{EIRP}{4\pi x^{2}}$$

According to the FCC’s relevant regulations on radiating antennas operating in the ISM band, the maximum power input to the transmitting antenna is limited to 30 dBm, and the *EIRP* cannot exceed 36 dBm. Assuming that the *EIRP* of Formula (4) reaches the maximum standard and that the transmitting mismatch loss is ignored, it can be stated that the radiation safety of the human body can be guaranteed when the distance *x* between the transmitting antenna and the receiving antenna is no less than 230 mm.

### 3.4. Wireless Power Transfer

**Figure 18:**
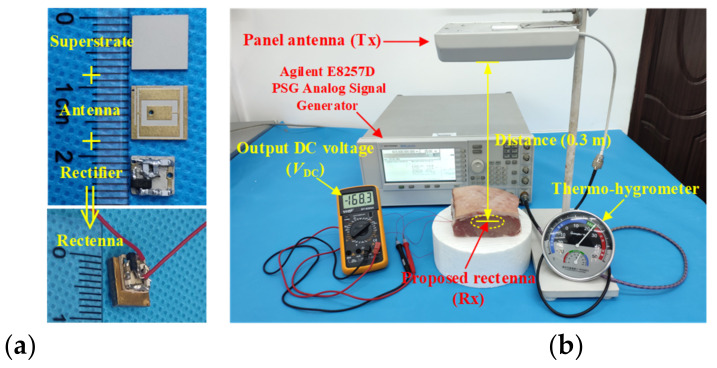
Photograph of measurement setup for wireless power transfer: (**a**) Fabricated implantable rectenna and (**b**) measurement setup.

**Figure 19:**
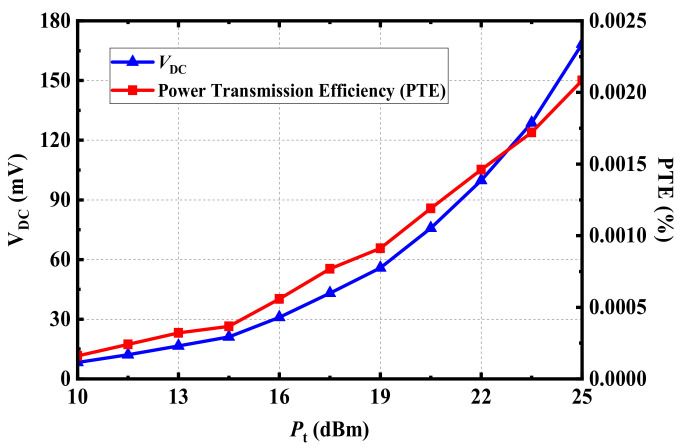
Measured output DC voltage and power transmission efficiency of the proposed rectenna at 915 MHz.

On the premise of meeting the radiation safety requirements discussed above, the performance of the rectenna was tested, as depicted in Figure 18. The selected distance between the transmitting antenna and the receiving antenna was 300 mm. In the RF energy-harvesting experiment, the proposed implantable rectenna was implanted in the muscle layer of fresh pork. The transmitting antenna working as the RF energy source was a panel antenna with a gain of 9 dBi. The transmitting antenna was vertically fixed at a height of 300 mm above the implanted rectenna and was connected to the RF power source by the coaxial cable. The RF power source was an analog signal with a frequency of 915 MHz, and the power was set as 25 dBm, meeting the *EIRP* standard of less than 36 dBm. At this point, the RF power received by the proposed rectenna could be converted to the DC voltage through its internal rectifier circuit. The output DC voltage measured by the voltage meter was 168.3 mV. In addition, the output DC voltage of the rectenna could be changed by adjusting the input power of the RF power source feeding to the transmitting antenna. Figure 19 shows the power transmission efficiency (PTE) evaluated by the output DC voltage *V*_DC_.

Considering the evaluation of the received power given in Figure 17, when the transmitting distance was 0.3 m, the possible received power of the proposed antenna was about –16.1 dBm, located in the range of between −15 dBm and −20 dBm. Furthermore, according to the simulated results of the rectifier circuit in Figure 16, when the RF power inputs to the circuit were −15 dBm and −20 dBm, respectively, the rectifier circuit could produce DC voltages of 190 mV and 81 mV on *R*_L_, accordingly. According to the measurement results from Figure 18b, the output DC voltage of the proposed rectenna was 168.3 mV, within the voltage range of 190 mV to 81 mV, indicating that the simulated result and measurement data were roughly consistent.

## 4. Conclusions

In this work, a broad dual-band implantable antenna for RF energy harvesting and data transmission was presented. The comparison of the proposed implantable antenna with current research is given in Table 3. Although the antenna in [24] has the smallest size, it has narrow bandwidth in two bands. Compared with the antennas in [22,23], its dimensions are all larger and its bandwidths are still small. The advantages of the proposed antenna are that its overall size is small and it achieves wider bandwidths in both desired bands. The 915-MHz ISM band can be used for RF energy harvesting, and the 2.45-GHz ISM band can be applied in data transmission. A sensitivity analysis of the proposed antenna was presented in detail. The performance of the proposed antenna was verified by actual measurement. Furthermore, an integrated rectenna scheme was proposed by employing a voltage-doubled rectifier circuit. The performance of the proposed implantable rectenna was evaluated and verified based on the 915-MHz ISM band for RF energy harvesting. With wide bandwidths and moderate dimensions, the proposed dual-band implantable antenna is applicable for telemetry medical equipment.

## Figures and Tables

**Table 1:** Optimized Antenna Dimensions (UNIT: mm).

Parameter	Value	Parameter	Value	Parameter	Value
*L*_1_	7.7	*L*_2_	7.9	*L*_3_	3.8
*L*_4_	4.95	*L*_5_	5.6	*L*_6_	6.8
*L*_7_	5.7	*w*_1_	0.55	*w*_2_	0.5
*w*_3_	0.2	*s*_1_	3.5	*s*_2_	3.65
*d*_1_	0.8	*d*_2_	0.15	*d*_3_	1.35
*h*	0.635	–	–	–	–

**Table 2:** Dielectric Properties of Three-Layer Human-Tissue Model.

Tissue	915 MHz		2.45 GHz	
	*ε_r_*	*σ* (S/m)	*ε_r_*	*σ* (S/m)
Skin	41.3	0.87	38.0	1.46
Fat	5.5	0.05	5.3	0.10
Muscle	55	0.95	52.7	1.74

**Table 3:** Performance Comparison with Previous Studies.

Ref.	Frequency (MHz)	Volume (mm^3^)	Bandwidth (|S_11_| < −10 dB)	Gain (dBi)	Implant Depth	Transfer Distance (mm)	Conversion Efficiency
[22]	402	16 × 14 × 1.27 (284.48)	8.4%	−35.9	10 mm muscle	500	51.7% at−5 dBm (one-series diode)
	915		5.7%	−24.3			
[23]	402	π × 5.5^2^ × 1.28 (121.6)	–	–	16 mm skin (with reflector)	200	68.9% at 30 dBm
	915		–	−23.2			
[24]	915	π × 4.8^2^ × 0.26 (9.4)	9.8%	−24.6	55 mm skin	60	50% at−14 dBm 76.1% at 2 dBm
	1470		7.34%	−18.3			
This Work	915	7.9 × 7.7 × 1.27 (77.25)	44.2%	−28.9	12 mm muscle	300	52% at 5 dBm
	2450		33.5%	−29.5			

## Data Availability

The Data presented in this study are available on request from the corresponding author. The data are not publicly due to potential patent application.
